# How Work Characteristics Are Related to European Workers’ Psychological Well-Being. A Comparison of Two Age Groups

**DOI:** 10.3390/ijerph15010127

**Published:** 2018-01-13

**Authors:** Laura Lorente, Núria Tordera, José María Peiró

**Affiliations:** 1 IDOCAL, University of Valencia, 46010 Valencia, Spain; nuria.tordera@uv.es; 2 IVIE & IDOCAL, University of Valencia, 46010 Valencia, Spain; jose.m.peiro@uv.es

**Keywords:** work characteristics, experienced meaningfulness, job satisfaction, psychological well-being, age

## Abstract

This study aimed to analyze the mechanisms through which work characteristics are related to psychological well-being, exploring the mediational role of work meaningfulness and job satisfaction, and investigating differences in the patterns of relationships between two age groups. The sample was composed of 36,896 workers from the 5th European Working Conditions Survey. Structural equation modeling analyses and multiple group analyses were performed. The results revealed a parallel mediational model, in which work meaningfulness and general job satisfaction mediate the relationships between work characteristics and well-being. Additionally, job satisfaction partially mediates the relationship between meaningfulness and well-being. These results were confirmed in both age groups (under 55 years old and older workers), but age moderates the relationships between social support and the mediating variables and the relationships between the mediating variables and general well-being. The present study uncovers significant pathways through which time pressure, decision latitude, and social support are related to psychological well-being, depicting an important step in better understanding how and when work characteristics are related to positive outcomes. It provides important clues for promoting psychosocial health at work at the European level.

## 1. Introduction

The European Commission [1], with its Europe Strategy 2020 to design jobs for a diverse workforce, aims to promote high quality, healthy jobs. This strategy is in line with developments in organizational health psychology designed to promote healthy workplaces and contexts where people can achieve high satisfaction and well-being, as well as high performance [2]. Work characteristics, when adequately designed and implemented in appropriate conditions, can provide meaning, satisfaction, and a sense of accomplishment and success; therefore, they play an important role in human well-being [3]. Nevertheless, workers can react differently to the same work environment factors depending on their age [4].

Thus, the main purpose of this study is to analyze the mechanisms through which work characteristics are related to psychological well-being. It focuses on the mediating role of meaningfulness and general job satisfaction in the relationship between three key work characteristics and psychological well-being. It also tests whether this model differs for older workers (who have upcoming retirement), compared to younger workers.

Different models have shown the importance of work characteristics in workers’ satisfaction and well-being [5]). Bourbonnais, Comeau and Vézina [6] pointed out that job demands of a quantitative nature (such as time pressure), decision latitude or control, and social support are the three most studied work characteristics in relation to health.

Regarding the mechanisms that link work characteristics to psychological well-being, the Job Characteristics model (JCM) [7] describes how core job characteristics are related to critical psychological states, and how these states, in turn, lead to positive outcomes. These propositions have been supported by meta-analytic results [8], pointing out that experienced meaningfulness is the “most critical” psychological state. Meaningfulness refers to the alignment between the demands of a person’s work role and his/her beliefs, values, and standards; it is the degree to which an employee feels the job has value. The specific characteristics of a job determine its experienced meaningfulness, which, in turn, contributes to work-related positive outcomes, such as motivation, performance, or job satisfaction [7]. Based on the JCM, we can expect experienced meaningfulness to be a key mediator between work characteristics and, job satisfaction and psychological well-being. Theories about general well-being consider the importance of experiences in different life contexts for its development. In this regard, some approaches, such as eudaimonic well-being, emphasize a meaningful work context as a central part of well-being. Therefore, we expect meaning to have a direct and positive relationship with general psychological well-being. Thus, we extend the JCM model by incorporating well-being in general, and not just related to the work context. In addition, we consider job satisfaction as an additional mechanism linking job characteristics to psychological well-being. The spill-over theory [9] suggests that feelings in specific dimensions of life affect feelings in other domains. This means that there could be a transmission of states of well-being from the work domain to general well-being. We argue that affective responses to work experiences (i.e., job satisfaction) will directly influence psychological well-being, as previous studies have shown [10]. Moreover, the influence of meaningfulness on well-being would be partially mediated by its relationship with job satisfaction.

**Hypothesis 1** **(H1).** The relationship between work characteristics and psychological well-being is mediated by experienced meaningfulness and general job satisfaction.

**Hypothesis 2** **(H2).** General job satisfaction partially mediates the relationship between experienced meaningfulness and psychological well-being.

Today’s societies are facing important demographic changes, one of them being the increase in the proportion of older persons and people aged 65 years or older who are still working throughout the European Union [11]. This change is reflected in events such as the declaration of 2012 as the “European year of active ageing and solidarity between generations”. Moreover, some studies suggests that there is a high probability that life expectancy will continue to increase in industrialized countries in the central and Western Europe [12]. This, together with the economic circumstances like the economic crisis, are contributing to the retention of older workers in workforces. Eurofound research reveals that employment of older workers in the European Union has increased in all types of jobs, and pensioners are increasingly involved in paid work [13]. Therefore, policies must be implemented to help people to stay healthy and active in working life during ageing [14]. Therefore, age has become an important variable for job design researchers and practitioners in promoting and maintaining workers’ well-being and health [15].

**Figure 1:**
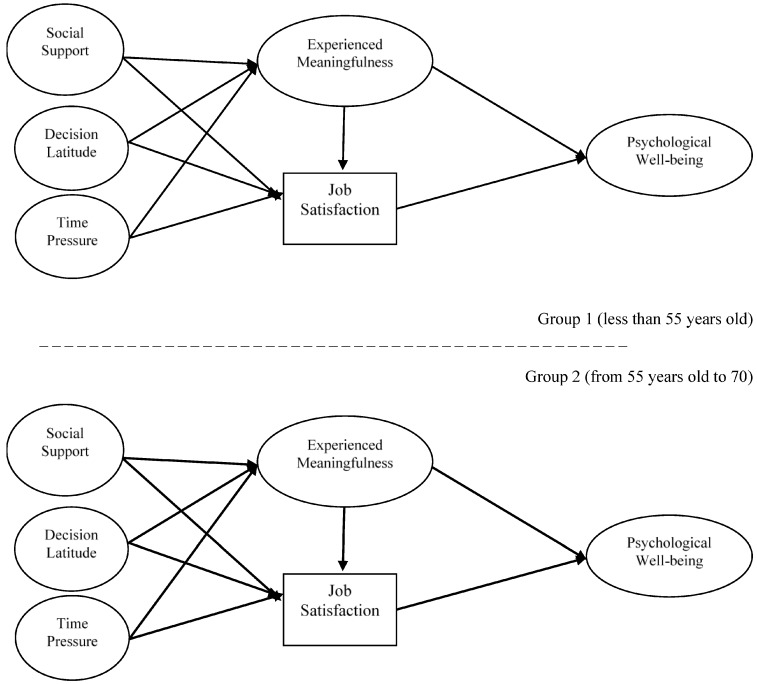
Hypothesized model for both age groups.

This study examines the moderator role of age in the patterns of relationships in the proposed model, presented in Figure 1.

There are several reasons for considering age as a moderator in the model. First, age is a covariate of different variables related to workers’ capabilities [4], and it has been related to job satisfaction, with more positive attitudes generally encountered in older workers [16]. However, these studies do not provide much information about the dynamics among work, age, and well-being [17]. Second, life span theories such as the Selection Optimization and Compensation model (SOC) and Socioemotional Selectivity Theory (STT) have acknowledged the impact of age on individuals’ preferences, goals, and motivations, and the way their fit with individuals’ actual circumstances might affect their satisfaction and well-being [4]. Both theories make similar predictions about differences in the goal representations of younger and older adults. More specifically, SOC proposes that, as individuals age, they will allocate fewer resources to growth objectives and more resources to maintenance and regulation of loss objectives. Thus, older workers could be expected to be more motivated toward work characteristics that allow them to achieve the maintenance and regulation of loss than the rest of the employees. STT theory proposes that, as individuals age, due to the changes in their future time perspective (more limited than their younger counterparts), they prioritize behaviors that provide them with meaning in life, a sense of belonging, and intimacy with others rather than behaviors related to learning, changing the environment, and advancing in their careers. Based on life span theories and the job characteristics theory, Truxillo and colleagues [4] formulated different propositions about how age would moderate the relationship between job characteristics and well-being. Following their propositions, we examine how age could moderate the relationship between work characteristics and psychological well-being. As Recher et al. suggest [11], ageing of the health workforce is a challenge, and policies need to be pursued in order to meet the particular needs of older workers. Therefore, taking into account the role of age in studies about health and well-being at work is necessary, to obtain realistic conclusions that allow the development of policies and interventions.

Autonomy has been found to have a more important impact on the health and well-being of older workers than on other workers, maybe because they may work autonomously due to their greater work experience and crystallized intelligence. Moreover, autonomy will allow them to select their accumulated skills and adapt their jobs in order to maintain their resources, as the SOC model proposes. Therefore, it is expected to be related to higher levels of meaningfulness, job satisfaction, and well-being.

The social support received from the supervisor and coworkers may be more attractive for older workers because, as STT Theory postulates, it could fulfill the desire for emotional intimacy and to feel more socially embedded within the organization. Thus, we expect the relationship between social support and well-being outcomes to be stronger for older workers. 

We expect work intensity to have negative effects on both age groups. However, increases in the pace at which employees have to do their work might pose a greater challenge to the abilities of older workers, due to the negative relationship between age and fluid intelligence [18]. 

To our knowledge, previous research has not addressed the moderator role of age in the relationship between these work characteristics and psychological well-being, nor has it taken into account the mediator role of meaningfulness and job satisfaction.

**Hypothesis 3** **(H3).** Age will moderate the paths of the parallel mediation model of experienced meaningfulness and job satisfaction between work characteristics and psychological well-being, so that the moderated hypothesized paths will be stronger for older workers than for the rest of the workers.

Two age groups are differentiated: younger than 55 years old and from 55 years old to 70. In this way, we aim to differentiate between people who have upcoming retirement (and therefore have a shorter future time perspective in work) and people who do not [4].

Summarizing, following robust theoretical models and based on data from a large harmonized European database, this study analyzes the mechanisms through which work characteristics are related to psychological well-being, exploring the mediational role of work meaningfulness and general job satisfaction, and investigating differences in the patterns of relationships between older and younger workers.

## 2. Materials and Methods

Data come from the 5th EWCS, conducted by the European Foundation for the Improvement of Living and Working Conditions [19]. The survey is based on a questionnaire administered face-to-face at home in the national language(s) of the country. It was applied to a random sample of “persons in employment” representative of the working population in 34 European countries (27 EU Member States, Turkey, Croatia, Republic of Macedonia, Norway, Albania, Kosovo and Montenegro). 

The basic sample is a multi-stage, stratified, random sample. Interviews were conducted among 43,816 respondents, yielding an overall response rate of 59.6% (for more details about the sampling design, see the methodology section of [19]. From the original sample, we excluded 15.8% of the respondents due to missing data. Our final sample was composed of 36,896 European workers with an age range of 15–70 years (M = 41.26, SD = 11.89). 16.2% were 55 years old or more. Fifty-two percent were male and 48.5% female. 

The work characteristics dimensions contain items that can be considered a proxy of Karasek’s Job Content Questionnaire (JCQ) because the items were modeled to make them comparable with previous results [10]. Time pressure was measured using two items. The alpha reliability for this scale is 0.78. Decision latitude was measured using five items corresponding to Karasek’s concept of “job control”. The alpha reliability for this scale is 0.77. Social support was measured using 2 items, with an alpha of 0.70. Experienced meaningfulness is a summative index created by adding two items, with an alpha reliability of 0.89, and shows a strong correlation with the Intrinsic Job Quality Index [20]. Job satisfaction was measured using a single item: “On the whole, are you very satisfied, satisfied, not very satisfied, or not at all satisfied with the working conditions in your main paid job?” Finally, psychological well-being was measured using the mental well-being index (WHO-5). The alpha reliability for this scale is 0.88. 

The statistical analyses included several steps. First, Intraclass Correlation Coefficients (ICC) were calculated to check that the data were pooled across countries. Specifically, ICC (1) estimates the proportion of variance between participants that could be accounted for by differences in group membership. Second, a principal components analysis was performed with all the items included in the study, using the Principal Axis Varimax Rotation procedure to test for the factorial structure of the scales. The factor analysis differentiated the dimensions of time pressure, decision latitude, social support, meaningfulness, and general well-being. Third, descriptive analyses and inter-correlations among the variables under study were calculated. Fourth, because data were all self-reported, there are concerns that the results might be influenced by common method variance, and so the common latent factor method (CLF) was implemented [21]. Finally, structural equation modeling implemented via AMOS 22.0 (SEM) was used to test the model, and multiple group analyses allowed us to analyze the invariance of the best model fit and investigate possible age differences in the patterns of relationships among the variables for two age groups. A statistical control was also used to reduce the risk that the relationships studied are the result of other confounding variables. After reviewing previous literature related to our study variables, education and income were also included in structural equation modeling as control variables because they could be affecting the other variables in our model. 

## 3. Results

### 3.1. Descriptive and Preliminary Analyses 

Means, standard deviations, and inter-correlations among the study variables are displayed in Table 1. 

Correlations were positive and significant, with effect sizes between moderate and large, except for time pressure, for which correlation was negative and small with job satisfaction and negligible with the other variables [22]. Thus, all the correlations were in the expected direction. The ICC (1) for the variables were: 0.06 for time pressure, 0.04 for social support, 0.03 for decision latitude, 0.05 for meaningfulness, 0.09 for job satisfaction, and 0.06 for psychological well-being; therefore, the values were not within the acceptable criterion for ICC (1) reported in previous reviews of multilevel research [23]. These results do not indicate an important role for the country in explaining the results. The CLF test produced a small change in the model fit [Delta χ^2^ = 3061, *p* < 0.001], which represented a slightly improved fit compared to the original model; however, when analyzing the significance of the structural parameters in both models (with and without the CLF), no significant changes in parameter estimates were found. These results indicate that the amount of variance due to common method bias is relatively small. 

### 3.2. Hypothesis Testing 

The SEM results are shown in Table 2. 

The first research model (M1) fits the data, and all the fit indices meet the criteria. All the path coefficients were significant, and these results confirm the parallel full mediation of experienced meaningfulness and job satisfaction between work characteristics and psychological well-being, confirming hypothesis 1. In the second model (M2), we added a path between meaningfulness and job satisfaction. In this way, we are assuming an additional effect of the sequential multiple mediation of experiential meaningfulness and job satisfaction on the relationships between work characteristics and general well-being. The model fit significantly improved, indicating that meaningfulness partially mediates the relationship between work characteristics and job satisfaction, and confirming hypothesis 2. However, an alternative model (M3) considered meaningfulness to be a full mediator rather than a partial mediator between work characteristics and job satisfaction, but the fit of the model (M3) deteriorated significantly. Finally, a fourth model (M4) was considered in which the direct effect from experienced meaningfulness to psychological well-being was not allowed, in order to analyze whether job satisfaction fully mediates between meaningfulness and well-being. Results showed than the fit of the model (M4) also deteriorated significantly.

In sum, the results show that the model with the best fit is model 2, as hypothesized. Paths are all significant, although effect sizes are variable, being smaller the effect of time pressure on the mediator variables and the effect of decision latitude on job satisfaction, while the rest are all between moderate and large [22]. This model shows that the relationships between work characteristics and psychological well-being are mediated in parallel by experienced meaningfulness and job satisfaction; in turn, experienced meaningfulness partially mediates between work characteristics and job satisfaction, and job satisfaction partially mediates between experienced meaningfulness and psychological well-being. The model explains 22% of the variance in job satisfaction and 19% of the variance in psychological well-being, and 47.3% of the effect of work characteristics on general well-being is mediated by the variables meaningfulness and job satisfaction. Regarding the control variables, after controlling for the effect of education and income on our independent, mediator, and dependent variables, the tested relationships were still found to play a significant role in the development of general well-being. Moreover, we found a significant effect of the two control variables on all the variables in the model, except for education on general well-being.

To test hypothesis 3, the invariance of M2 across two age groups was investigated, following the procedure recommended by Byrne [24]. The group of younger people (less than 55 years old) was composed of 31,089 employees, and the older group (55 years old and up, who are supposed to retire soon), was composed of 5807 employees.

**Figure 2:**
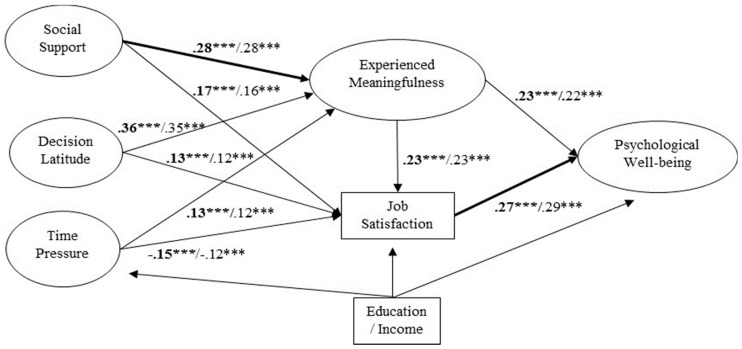
The best fitted model (M_2_) with standardized paths coefficients representing the relationships significantly stronger in the older group of workers. The figure includes results with and without the control variables (with the control variables in bold). *** *p* < 0.001. Note: Education and Income are control variables. In the original structural equation model computed by AMOS, both control variables presented paths to all variables in the model.

The fit of the fully constrained model deteriorated significantly [Delta χ^2^ = 135.5, *p* < 0.001], meaning that the structural weights of M2 are not invariant across the two age groups, as presented in Figure 2. Thus, age moderates the relationships in our theoretical model. Then, in order to identify which relationships showed differences between groups, we proceeded to release each parameter, comparing the fit with M2. Results showed that differences were identified in the relationships between social support and job meaningfulness [Delta χ^2^ = 5.86, *p* < 0.05], social support and job satisfaction [Delta χ^2^ = 9.82, *p* < 0.001], meaningfulness and psychological well-being [Delta χ^2^ = 4.45, *p* < 0.05], and job satisfaction and psychological well-being [Delta χ^2^ = 24.31, *p* < 0.001]. Specifically, these relationships were positive in both groups, but whereas the relationships between social support and meaning and between job satisfaction and psychological well-being were stronger in the older group, the relationships between social support and job satisfaction and between meaningfulness and psychological well-being were stronger in the group of younger workers. These results partially confirm hypothesis 3 because age moderates some, but not all of the relationships in the model. In fact, the relationships between autonomy and work intensity and meaningfulness and job satisfaction do not present differences between both groups, so we could conclude that autonomy is not more important for older workers nor that the work intensity is worse for them.

## 4. Discussion

Using a large and representative sample of European workers, this study has provided unique empirical support for a comprehensive model, identifying experienced meaningfulness and general job satisfaction as psychological states that mediate the relationships between work characteristics and employees’ psychological well-being. Moreover, significant moderating effects of aging are also found on a number of paths, especially the influence of social support on well-being.

The different work characteristics play different roles. Whereas job characteristics that represent work resources (decision latitude and social support) have positive relationships with both of the psychological states considered (meaningfulness and job satisfaction), the characteristic that represents demands (time pressure) presents negative relationships with both of these states. These results provide insights for redesigning work characteristics: Increasing decision latitude and social support may improve both experienced meaningfulness and job satisfaction, as well as psychological well-being. Experienced meaningfulness is a significant path in the hypothesized relationships [25], but it is not the only one because general job satisfaction also conveys the relationship between work characteristics and psychological well-being. This result agrees with previous results showing that job satisfaction is an important variable in general well-being beyond the work sphere [26]. Interestingly enough, job satisfaction partially mediates the relationship between experienced meaningfulness and psychological well-being, presenting a sequential partial mediation of job characteristics on well-being. 

As noted above, meaningful work experience is a significant conveyor in the positive relationship of job resources and the negative relationship of job demands with general job satisfaction and psychological well-being. The fact that meaningfulness has a positive and significant relationship with both outcomes corroborates some previous studies that noted its significant and positive relations with employee outcomes [27,28]. Experienced meaningfulness of work is not only important for work-related outcomes such as job satisfaction, but also for the general psychological well-being of workers. Job satisfaction opens up another parallel avenue through which work characteristics are related to psychological well-being, and it partially mediates the relationship between meaningful work and general well-being. These findings highlight the importance of work in other life spheres, supporting the spill-over theory [9], because positive psychological states at work have spillover effects on other life spheres, influencing general psychological health, and not just the work sphere, corroborating some previous research [10].

Finally, the results obtained have shown interesting specificities for the two age groups on the paths leading from social support through the mediating variables to well-being. Social support has a stronger relationship with work meaningfulness in older workers. However, it has a stronger relationship with job satisfaction in the younger group. These results show support for lifespan theories. Social support could be more meaningful for older workers because, through it, they fulfill the desire for emotional intimacy and to feel more socially embedded within the organization [29]. However, the fact that social support at work is usually directed toward enhancing employees’ skills or balancing their work-life responsibilities would make this characteristic more beneficial for the group of younger workers. Through social support, they would fulfill their need to strive to build knowledge or reduce the strain related to balancing work and family responsibilities [30]. In addition, for the older workers’ general well-being, job satisfaction is more important, whereas for the younger workers’ general well-being, experienced meaningfulness is more important. Again, this result supports life span theories. The different time perspective in older and younger workers changes their goals and affects the salience of different types of experiences for their well-being. The shorter time perspective of older workers makes job satisfaction more important for their well-being, whereas the longer time perspective of younger employees makes meaningfulness more salient.

## 5. Limitations and Future Research

The main limitation of the study is related to fact that all the data were gathered cross-sectionally from a single source. With regard to the use of self-reported measures, we used the CLF method, and the results reveal that common method variance is not necessarily a serious deficiency in this dataset. In addition, with the type of constructs considered, in most cases self-report is required as a way to access their nature (meaningfulness, job satisfaction, etc.). Moreover, taking into account the cross-sectional design used in this study and the complexity of the interrelations among the variables, a cause-effect relationship should not be inferred from these findings. Indeed, reversed relationships could also be produced between our independent and dependent variables. In this line, according to the to the assimilation process (imposition of internalized structures onto the external world) of Bless and Flieder [31], individuals with positive affect are also likely to attribute positive qualities to their job environments. For instance, people with higher levels of well-being could be more ready to do a positive evaluation of the job characteristic considered in this study (i.e., social support, decision latitude and time pressure). One may expect that causality direction could be tested by comparing the model fits of alternative SEM with arrows pointing in different directions. However, recent research shows that this is not a valid test of these alternative possibilities. Thus, although our results suggest support for the specified causal mechanisms, supporting previous cross-sectional and longitudinal studies [32], other causal sequences cannot be ruled out. 

Regarding job satisfaction, it was measured using a single item. However, mono-item measures of some variables such as job satisfaction have been considered reliable in industrial and organizational psychology [33].

Future research could test these relationships using longitudinal designs that may further clarify the dynamic of changes in job characteristics over time and the way these changes might influence the evolution of well-being at work and in general, based on a recent study that analyzed changes in working conditions and physical health functioning [34]. In addition, it would also be interesting to analyze the reverse causality, that is, how changes in well-being may affect the perception of work characteristics. Moreover, future studies could explore other job characteristics or psychological mechanisms that could have a differential impact on older workers’ well-being, for example taking into account distinct organizational change contexts [35].

## 6. Conclusions

This study represents an important step forward in understanding how and when work characteristics are related to general job satisfaction and psychological well-being. The “how” was clarified by identifying that work meaningfulness and job satisfaction represent two parallel and cumulative avenues through which work characteristics are related to psychological well-being. The “when” has been clarified by pointing out that the relationships between social support and meaningfulness and between job satisfaction and general well-being are stronger for older workers (i.e., when retirement is approaching).

From a Positive Psychology perspective, this study provides some hints about how to improve levels of meaningfulness and job satisfaction. In designing jobs while taking workers’ ages into account, organizational strategies should try to maintain adequate levels of time pressure at work and promote decision latitude and social support for workers of all ages. However, social support was found to be especially important for older workers. The present study also offers further insight on the different mechanisms linking job characteristics to well-being for different age groups.

## Figures and Tables

**Table 1:** Means (M), standard deviations (SD) and inter-correlations.

	M	SD	Response Scale	1	2	3	4	5	6
1 Social support	3.84	1.02	1–5	1					
2 Decision latitude	3.06	1.08	1–5	0.33 **	1				
3 Time pressure	3.57	1.85	1–7	−0.04 **	−0.04 **	1			
4 Meaningfulness	4.24	0.82	1–5	0.30 **	0.35 **	−0.11	1		
5 Job Satisfaction	2.96	0.74	1–4	0.26 **	0.23 **	−0.14 **	0.31 **	1	
6 Gen. Well-being	4.23	1.06	1–6	0.23 **	0.19 **	−0.04 **	0.28 **	0.36 **	1

Note: N = 36,896. ** *p* < 0.01.

**Table 2:** Model fit.

Model	χ^2^	df	RMSEA	SRMR	AGFI	CFI	IFI	Δχ^2^	Δdf
M1	19,322.1	134	0.062	0.06	0.92	0.91	0.91		
M2	18,203.6	135	0.060	0.06	0.92	0.92	0.92	M2 − M1 = 11,218	1 ***
M3	19,659.2	136	0.062	0.07	0.92	0.91	0.91	M3 − M2 = 1455.6	3 ***
M4	19,521.2	134	0.062	0.08	0.92	0.91	0.91	M4 − M3 = 138	2 ***

Note: N = 36,896. *** *p* < 0.001. χ^2^ = Chi-square; df = degrees of freedom; RMSEA = Root Mean Square Error of Approximation; SRMR = Standardized Root Mean Square Residual; CFI = Comparative Fit Index; IFI = Incremental fit index; M1 = Double mediation model, M2 = Hypothesized model (path from meaningfulness to job satisfaction), M3 = Alternative model (Full mediation of meaningfulness), M4 = Second alternative model (Full mediation of job satisfaction).
